# Important Metallurgical Discoveries of a Physician

**Published:** 1887-07

**Authors:** 


### Important Metallurgical Discoveries of a Physician.

Dr. C. C. Carroll, of Meadville, Pa., after years of experimenting, has discovered a method by which aluminum can be cast, soldered, and welded. It is claimed by metallurgists and artisans that this is a very valuable invention, since it insures the use of aluminum for many purposes on account of its extreme lightness, strength, and non-oxidation by exposure. It is already successfully employed in the manufacture of dental plate, for which it is apparently admirably adapted. In the course of his experimenting, Dr. Carroll believes he has also discovered the law governing the disintegration of iron stringers employed in the construction of railroad bridges.

Powdered Rice as a Styptic.—According to the India Medical Gazette, powdered rice has marked hæmostatic properties. It is said that if mixed with lint in proportion of from ten to eleven per cent., the lint thus treated being used as a compress, it is more effectual than oxide of lime, subnitrate of bismuth, salicylic acid, or carbolic acid.

